# Delivering Digital Healthcare for Elderly: A Holistic Framework for the Adoption of Ambient Assisted Living

**DOI:** 10.3390/ijerph192416760

**Published:** 2022-12-14

**Authors:** Manal Almalki, Majid H. Alsulami, Abdulrahman A. Alshdadi, Saleh N. Almuayqil, Mohammed S. Alsaqer, Anthony S. Atkins, Mohamed-Amine Choukou

**Affiliations:** 1 College of Public Health and Tropical Medicine, Jazan University, Jazan 45142, Saudi Arabia; 2 Applied College, Shaqra University, Shaqra 11961, Saudi Arabia; 3 College of Computer and Engineering, University of Jeddah, Jeddah 23218, Saudi Arabia; 4 College of Computer and Information Sciences, Jouf University, Sakaka 72388, Saudi Arabia; 5 College of Computer Science, King Khalid University, Abha 62529, Saudi Arabia; 6 School of Computing and Digital Technologies, Staffordshire University, Stoke-on-Trent ST4 2DE, UK; 7 Department of Occupational Therapy, College of Rehabilitation Sciences, University of Manitoba, Winnipeg, MB R3T 2N2, Canada

**Keywords:** Ambient Assisted Living, AAL technology, adoption, framework, elderly ageing in place

## Abstract

Adoption of Ambient Assisted Living (AAL) technologies for geriatric healthcare is suboptimal. This study aims to present the AAL Adoption Diamond Framework, encompassing a set of key enablers/barriers as factors, and describe our approach to developing this framework. A systematic literature review was conducted following the Preferred Reporting Items for Systematic Reviews and Meta-Analyses (PRISMA) guidelines. SCOPUS, IEEE Xplore, PubMed, ProQuest, Science Direct, ACM Digital Library, SpringerLink, Wiley Online Library and grey literature were searched. Thematic analysis was performed to identify factors reported or perceived to be important for adopting AAL technologies. Of 3717 studies initially retrieved, 109 were thoroughly screened and 52 met our inclusion criteria. Nineteen unique technology adoption factors were identified. The most common factor was privacy (50%) whereas data accuracy and affordability were the least common factors (4%). The highest number of factors found per a given study was eleven whereas the average number of factors across all studies included in our sample was four (mean = 3.9). We formed an AAL technology adoption framework based on the retrieved information and named it the AAL Adoption Diamond Framework. This holistic framework was formed by organising the identified technology adoption factors into four key dimensions: Human, Technology, Business, and Organisation. To conclude, the AAL Adoption Diamond Framework is holistic in term of recognizing key factors for the adoption of AAL technologies, and novel and unmatched in term of structuring them into four overarching themes or dimensions, bringing together the individual and the systemic factors evolving around the adoption of AAL technology. This framework is useful for stakeholders (e.g., decision-makers, healthcare providers, and caregivers) to adopt and implement AAL technologies.

## 1. Introduction

The majority of countries around the world are confronted with a rapidly ageing population. The number of people aged 60 years or older will be nearly doubled by 2050, rising from 1 billion in 2020 to 2.1 billion in 2050, and this figure represents approximately one-quarter (22%) of the total global population [1]. In older age, people are at high risk of comorbidity and are vulnerable to loss of vision, hearing and mobility, and non-communicable diseases such as heart diseases, chronic respiratory disorders, and dementia [1]. Falls are one of the common and serious health problem that people aged 60 years and older face which lead to fractures, injuries, loss of confidence, depression, and sometimes death [2,3]. Every year, over 300,000 elderly people are admitted to hospitals with hip fractures due to falling sideways [3]. Conventional methods of caring for people aged 60 years or older impose enormous stresses on their health caregivers. Caregivers’ ability to provide appropriate care for those older persons may be directly impacted by a variety of problems, such as a lack of health information and distance restrictions [4].

Intelligent systems and Ambient Assisted Living (AAL) technologies can help elderly remain safe and self-dependent, which could ease the stress on their caregivers [5,6]. AAL technologies have been developed by utilizing smart devices and techniques which include smart gadgets, wireless networks, software, and medical sensors [7]. Examples of AAL technologies are assistive robotics for sound-based activity monitoring, fall detection, and rescue include: sensor-based networks for controlling heating and air conditioning system at homes based on the weather conditions; wearable e-textile technologies for treating insomnia disorders by controlling body temperature during sleep [8]; and health phone-based apps and wearable devices such as smartwatches and bracelets for tracking vital signs like blood pressure, blood sugar, and heart rate. AAL technology enables tracking health information, empowering people to have more control over their health by keeping themselves informed about their physical functioning and health state. It also makes it possible for older people and their caregivers to identify health issues early and react to medical emergencies faster [9,10]. 

However, AAL technologies have not been broadly implemented to complement the conventional methods of taking care of the elderly [11,12]. Developing AAL technology-enabled healthcare services that are accepted and used effectively by the elderly is a complex process because it involves many inter-related factors such as individual and family involvement [7], private and public institutions [13], and technology [14] as a support for interventions. Smit and Eybers (2022) [14] gathered an unexhaustive list—and provided a critique—of theoretical frameworks on technology adoption, including the technology acceptance model (TAM) [15] the theory of planned behaviour (TPB) [16] the theory of reasoned action (TRA), and the technology-organisation-environment (TOE) framework [17], and found that these technological adoption theoretical frameworks are either focusing on the individual level or on the organisational level. For example, the TOE framework [18] explains technology adoption in organisations and illustrates how technological context, organisational context, and environmental context influence the process of adopting and implementing technological advances.

The current frameworks for technology adoption allow for a siloed view of the technology implementation process and do not allow for a thorough understanding of technology implementation within a health ecosystem. Furthermore, current frameworks are not necessarily centered on AAL. Although AAL technology offers promising autonomous healthcare for senior citizens and substantial assistance “for a better, healthier and safer life” [19], its adoption remained one of the biggest barriers [11,20]. Therefore, a holistic framework for the adoption of AAL is needed [21]. This study aims to 1. systematically review the literature on the adoption of Ambient Assisted Living (AAL) technologies for providing health care for the elderly and thematically analyze the relevant papers to identify enablers/barriers or factors contributing to the adoption of these technologies and 2. to propose a novel AAL technology adoption framework that consists of the retrieved factors. We believe such a framework would provide insight for stakeholders (e.g., decision-makers, healthcare providers, and caregivers) to maximize the adoption of intelligent systems and AAL technologies for delivering healthcare to older adults.

## 2. Materials and Methods

**Figure 1:**
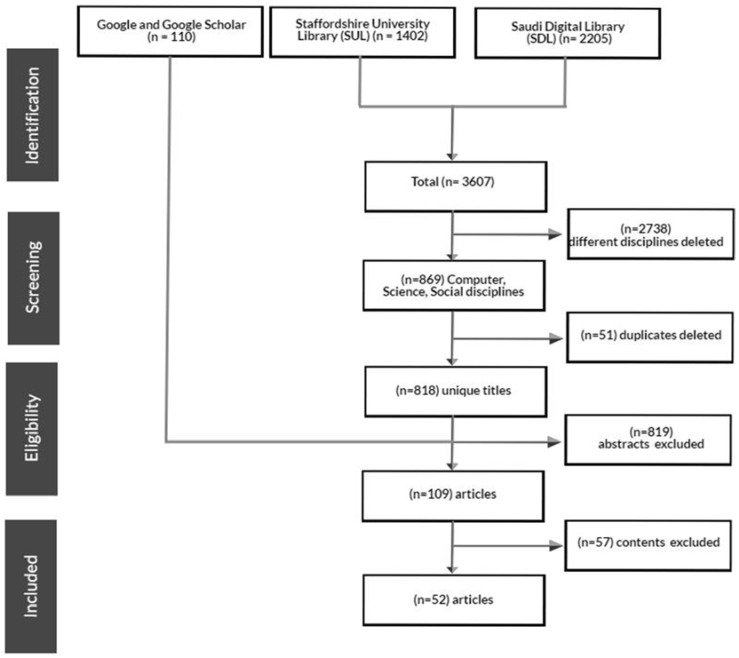
PRISMA Flowchart, adapted from Liberati et al. (2009) [22].

A systematic literature review was conducted using PRISMA (Preferred Reporting Items for Systematic Reviews and Meta-Analyses) guidelines [22,23]. Figure 1 presents a flow chart illustrating the review procedures. 

### 2.1. Review Procedures

Step 1: Identification. 

Saudi Digital Library (SDL) and Staffordshire University Library (SUL) that include databases such as SCOPUS, IEEE Xplore, PubMed, ProQuest, Science Direct, ACM Digital Library, SpringerLink, and Wiley Online Library were searched to find relevant publications. Google (e.g., working papers and websites) and Google Scholar were also searched. The keywords used to find respective publications were as follows: ((((“ambient assisted living”) OR (“smart home*”) OR (“assistive robotic*”) OR (“assistive robot*”) OR (“wearable and mobile device*”) OR (“e-textile*”)) AND ((“older people”) OR (“senior people”) OR (“elderly population”) OR (“ageing population”) OR (“aging population”) OR (“population ageing”) OR (“elderly person*”) OR (“older person*”) OR (“elderly people”)) AND ((issue*) OR (barrier*) OR (factor*) OR (challenge*)))).

Step 2: Screening.

A total of 3717 studies were initially retrieved: 3607 from SDL and SUL, and 110 from Google and Google Scholar. Duplicated papers were identified and then removed using Refworks.proquest.com that checked the similarity of titles and year of the publication. Next, titles, abstracts, and keywords were screened. 

Step 3: Eligibility.

Any papers or reports that met the following criteria were considered eligible for inclusion as illustrated in Table 1.

Step 4: included Studies.

After excluding different disciplines and duplicated papers, 109 studies were thoroughly screened, and 52 studies were eligible for inclusion, as shown in Figure 1. The first, second, and third authors examined the content of each of the selected articles, applying the inclusion criteria. If the authors did not reach an agreement, the fourth and fifth researchers could decide. Disagreements about the inclusion of any article were resolved by consensus.

### 2.2. Thematic Analysis Procedures

Thematic analysis was performed to examine the included studies and identify themes pertaining to possible enablers/barriers or factors contributing to adopting AAL technology. Our thematic analysis involves six phases described by Braun and Clarke (2006) [24]: familiarizing yourself with your data, generating initial codes, searching for themes, reviewing themes, defining and naming themes, and producing the report.

The selected articles were read and initial ideas for coding were developed. Codes were manually generated by highlighting and writing them in Microsoft Word, which represents factors reported or perceived to be important by authors of the included studies. Following this, similar codes were grouped to form overarching themes. After that, codes were compared to check consistency and cohesion within groups and across categories or groups (i.e., there were no codes that could equally fit within two different groups). Themes were named dimensions, and codes were named factors. Finally, tables and pictures were used to report the findings and demonstrate the process for producing concise, coherent, logical and non-repetitive themes; dimensions; sub-themes; and factors as suggested by Braun and Clarke (2006) [24]. 

## 3. Results

A total of 52 publications were eligible for inclusion in this study, and 19 unique factors were identified as a result of the thematic analysis.

### 3.1. Characteristics of the Included Studies 

Table 2 presents the included publications in an ascending order from the oldest to most recent by publication year and summarizes their characteristics. The table shows the first author’s name, year of publication, and different types of technologies used for providing health care to the elderly: Ambient Assisted Living (AAL) had 23 articles, Smart Homes (SHs) had 15 articles, Assistive Robotics (AR) had 7 articles, Wearable and Mobile Devices (WMD) had 5 articles, and e-Textiles (e-T) had 2 articles.

### 3.2. Our Approach Establishing the Framework of AAL Adoption

**Figure 2:**
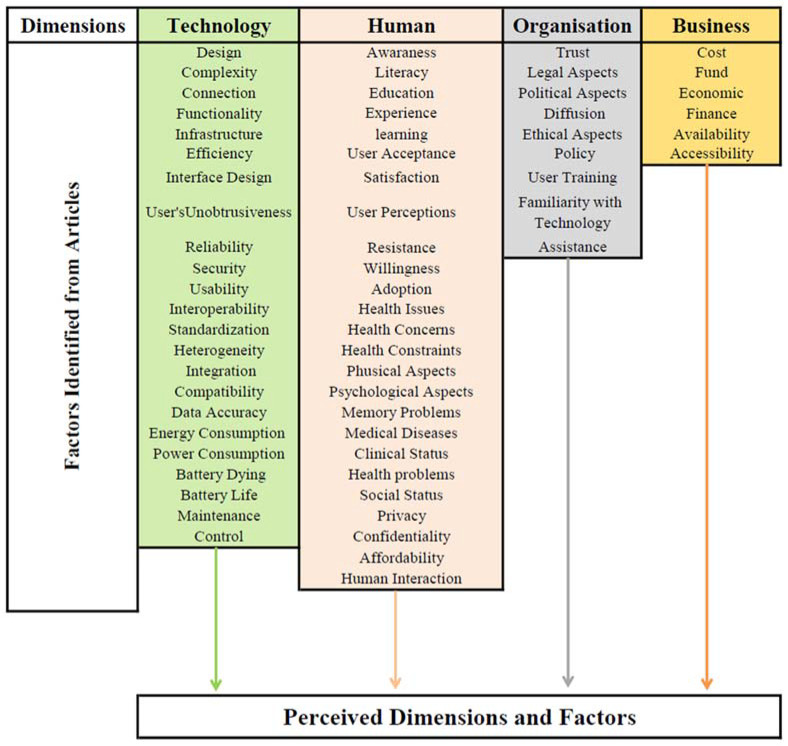
Map of initial dimensions and factors.

Iterative revision of codes/factors and themes/dimensions was conducted to establish the intended framework. In the initial rounds, sixty-three were identified based on how frequently they appeared in our sample of articles, and then grouped under four dimensions: Technology, Human, Organisation, and Business, as shown in Figure 2.

**Figure 3:**
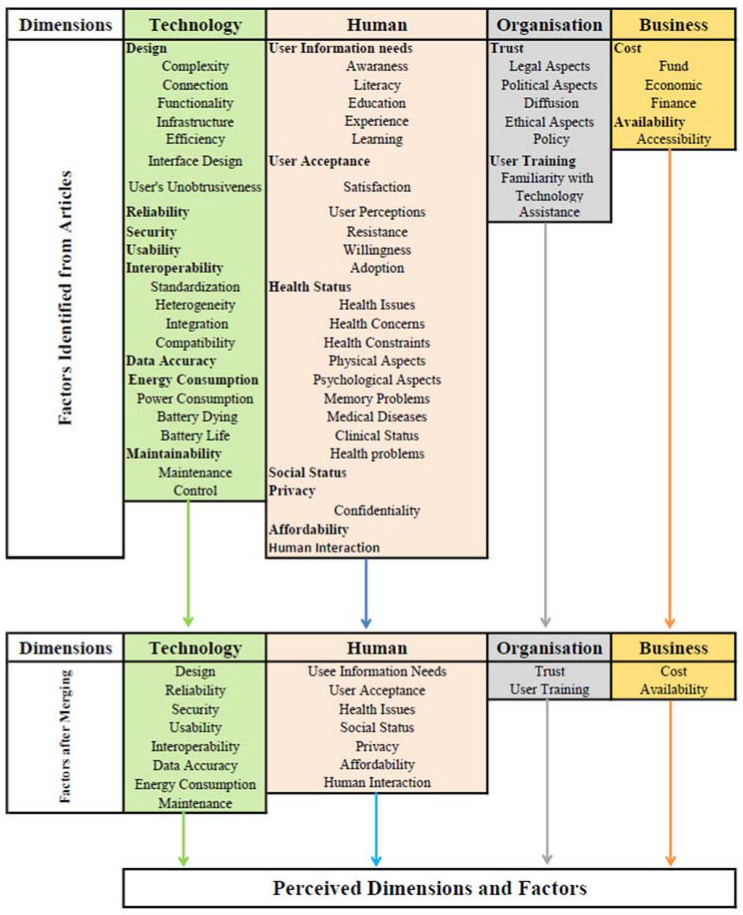
The dimensions and factors after revision.

After iterative reviews and refinements of codes and themes, the four dimensions were retained in the final rounds, but the total number of factors was reduced to nineteen (Figure 3). The factors combined and/or retained were as follows:

- Technology dimension has eight factors:
  ∘ Design, complexity, connection, functionality, infrastructure, efficiency, interface design, and user’s unobtrusiveness were abstracted and placed under “design” factor.
  ∘ Interoperability, standardization, heterogeneity, integration, and compatibility were grouped and placed under the interoperability factor.
  ∘ Energy consumption, power consumption, battery dying, and battery life were grouped and renamed as energy consumption factor.
  ∘ Maintenance and control were grouped and renamed maintainability.
  ∘ Reliability, security, usability, and data accuracy factors were all retained with no changes.
- Human dimension has seven factors:
  ∘ Awareness, literacy, education, experience, and learning were abstracted and renamed as user’s information needs.
  ∘ User Acceptance, satisfaction, user perceptions, resistance, willingness, and adoption were grouped and renamed as user acceptance factor.
  ∘ Health issues, health concerns, health constraints, physical aspects, psychological aspects, memory problems, medical diseases, clinical status, and health problems were grouped and placed under health status factor.
  ∘ Privacy and confidentiality were grouped and renamed as privacy factor.
  ∘ Social status, affordability, and human interaction were all kept with no change.
- Organisation dimension has two factors:
  ∘ Trust, legal aspects, political aspects, diffusion, ethical aspects, and policy were abstracted and placed under trust factor.
  ∘ User training, familiarity with technology, and assistance need were grouped and renamed as user training factor.
- Business dimension has two factors:
  ∘ Costs, funds, economic, and finance were grouped and renamed as costs factor.
  ∘ Availability and accessibility were grouped and renamed availability factor.

### 3.3. Key Factors Contributing to AAL Technologies Adoption

The thematic analysis of the included studies led to identifying nineteen unique factors as listed below. The most common factor was privacy (50%), whereas the least common factors, 4% each, were data accuracy and affordability. These factors are listed in a descending order based on their frequency in our sample of studies included.

1. Privacy factor was noted in 50% of the included studies. It refers to the privacy of AAL users’ personal information. Poor privacy of AAL technologies can cause invasion of users’ private lives and leads to refusal of adoption by users [11].
2. Costs factor was noted in 46% of the included studies. It includes expenses such as purchasing, installation, and maintenance fees [11,56].
3. Trust factor was noted in 44% of the included studies. It indicates a lack of trust and acknowledgement of organisations and ambient towards technologies [11].
4. Security factor was noted in 40% of the included studies. It indicates secure communication among main components of AAL technologies [64]. Studies suggested that new technologies should be designed with security measures taken into consideration in order to increase AAL technologies’ adoption among older adult users [59].
5. Design factor was noted in 35% of the included studies. It is concerned with the constructability of AAL technologies that includes, but not limited to, complexity, connection, functionality, infrastructure, efficiency, interface design, and user’s unobtrusiveness. Studies suggested that aging users should be engaged in designing new technologies to enhance their design [65].
6. Interoperability was noted in 27% of the included studies. It refers to the ability of different systems to communicate with each other to provide the intended services. Lack of interoperability among AAL devices used by older adult users could hinder its long-term adoption [59].
7. User’s information needs factor was noted in 23% of the included studies. It indicates that AAL technologies’ capability to fulfill the users’ information needs, which may differ based on several elements such as the user’s health status, literacy, technical skills and the like.
8. User acceptance factor was noted in 23% of the included studies. It refers to the acceptance of AAL technologies by its perceived users [11]. The acceptance of AAL technologies varies for different age groups and influences many aspects such as ease of use.
9. Social status factor was noted in 23% of the included studies. It is related to understanding the gender position that older persons hold in a group (e.g., grandfather, unmarried) and the impact of AAL technologies on their social activities. According to Wu et al. (2014) [43], AAL technologies could reduce communication between the users and their family members.
10. Health status factor was noted in 19% of the included studies. It refers to providing elderly people with technologies that can help them monitor their health and understand users’ wellness and illness including physical disabilities, chorionic diseases, mental impairments, etc. [45].
11. User training factor was noted in 15% of the included studies. It refers to providing the ability for older users to use AAL technologies through training and customer services to enhance their autonomy in human-free assistance [63].
12. Usability factor was noted in 15% of the included studies. It is concerned with the older users’ ability and desire to use the AAL technology. Limited ability of the older adults could be due to several factors such as lack of confidence in using new technologies [65].
13. Reliability factor was noted in 13% of the included studies. It can be described as the possibility of the technology to provide its perceived benefits. Poor reliability of AAL technologies can lead to low utilization by users [59].
14. Availability factor was noted in 13% of the included studies. It indicates the availability of AAL technologies and services to consumers in the required or local markets [11] despite any change in the company or service provider.
15. Energy consumption factor was noted in 12% of the included studies. It is concerned with efficiency in energy usage. Low energy consumption of AAL technology reduces users’ expenses and improves their usage [64].
16. Human interaction factor was noted in 12% of the included studies. It is concerned with the interaction between the devices and their users to get the functions completed. Here the balance of manual functions that must be performed by the users and automatic functions that are performed by the devices should be maintained for effective human interaction and engagement of the older users [65].
17. Maintainability factor was noted in 10% of the included studies. It refers to the capability of maintaining the AAL system and keeping it up to date. Maintainability is essential for a long-term adoption [63].
18. Affordability was noted in 4% of the included studies. It refers to whether the price of the AAL technology is within most ageing consumers’ budget [54].
19. Data accuracy factor was noted in 4% of the included studies. It refers to the correctness of data values produced by AAL technologies with high accuracy [55].

## 4. Discussion 

**Figure 4:**
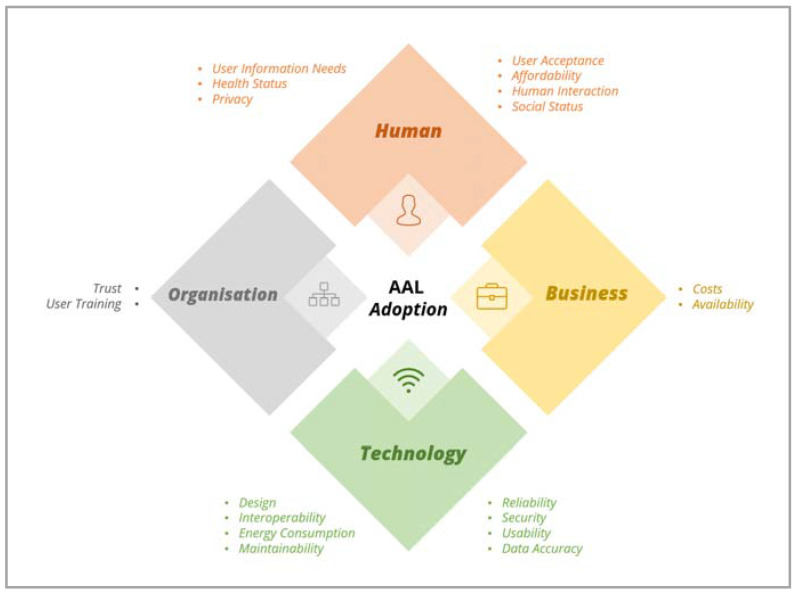
AAL Adoption Diamond Framework.

The goal of this study was to systematically review the literature on the adoption of AAL technologies for providing health care for the elderly with the intention of identifying the key enablers and barriers to the adoption of AAL technologies to propose a novel AAL technology adoption framework. Thematic analysis of the included studies was performed, and iterative grouping and refinement of codes/factors and themes/dimensions were undertaken. Nineteen factors and four dimensions were identified, leading to the formation of the AAL Adoption Diamond Framework (Figure 4). The following sections discuss these dimensions and factors and compare the results of our study with the included ones. 

### 4.1. The AAL Adoption Diamond Framework

The AAL Adoption Diamond Framework recognizes nineteen factors and organises them into four dimensions: Human, Technology, Business, and Organisation. The factors identified were considered important constituent of the Diamond Framework due to their contribution to AAL technologies adoption. For example, the factors privacy, costs, trust, security, and design were the most frequent factors (reported in more than one-third of the included studies), and significantly affected the adoption of AAL technologies. Poor protection of personal information can cause invasion of the user’s private life and, in turn, lead to hindering the adoption of these AAL technologies [11]. Similarly, insecure systems allow breaches to users’ privacy and could hinder the users’ desire and obstruct the adoption of these technologies [64]. Lack in trust of the organisations that made the AAL technology is a problem that can impede the decision of its adoption (4, 13, 20). For costs, the price of AAL technologies was seen to be a barrier to purchasing by older persons if it was high [54].

On the other hand, data accuracy and affordability were the least frequent factors in our review, these factors have a significant impact on the decision to adopt AAL technologies. Data gain its values from being correct and consistent [55]; thus, poor data accuracy leads to low user acceptance and implementation, hence obstructing the AAL technologies adoption. Likewise, AAL technologies whose price is not affordable by the older customers [54] could negatively affect its adoption. 

### 4.2. Comparison between Findings from the Included Studies and Our Study

Overall, the findings of our systematic review suggest that included studies did not optimally recognize all key factors. Table 1 illustrates the number of factors identified by the included studies, which were also organised per our four dimensions. In our study, a list of factors was identified that is holistic in terms of their number and novel and unmatched in terms of classifying them into four categories or dimensions and presenting them in the AAL Adoption Diamond Framework. For example, nineteen factors were described in our study, whereas, in our sample of included studies, the average number of factors identified was about four (mean = 3.9). A total of 22 studies (42.3%) noted only two factors, and almost one-third (32.7%, N = 17) of the included studies reported three to five factors. The highest number of factors was eleven, and it occurred in one study by Grgurić (2012) [29]. Ten factors were found in two studies [45,63].

In addition, 17.3% and 7.7% of the included studies pointed out 6–8 factors and 9–11 factors, respectively. However, these factors were not structured to show the relations among them in how it was performed in our work. The AAL Adoption Diamond Framework holistically describes the nineteen factors and organises them into four key dimensions based on their relation and contribution to the corresponding dimension.

### 4.3. Comparison between Our AAL Adoption Diamond Framework and Popular Technology Adoption Models and Frameworks

In term of AAL design, AAL models and frameworks must be built around the users’ needs and expectations and this was considered during the development of the AAL Adoption Diamond Framework. However, most AAL models and frameworks are not designed to inform the developers’ or geriatricians’ nor give them insights on ambient needs of the older adults; and this could lead to failure in achieving a long and/or even short-term adoption of AAL [65]. For so long, the end-user has only been regarded from one perspective: either that of the patient in patient-focused models (such as the TAM) or that of the institution in system-oriented models (e.g., the TOE framework). In practice, analysis should methodically concentrate on the micro, mezzo, and macro levels, i.e., the people (patients, families, professionals), the institutions (clinics, hospitals, insurances, community), and the healthcare system (health sector within a given jurisdiction).

In terms of the model’s depth and holism, our AAL Adoption Diamond Framework identifies four key dimensions and nineteen factors. These factors can be further assessed through examining a set of concepts and aspects as illustrated in Figure 2. This depth evaluation could play an important role in making informed decisions about the design of ambient technologies for elder people, and hence improve the chance of their adoption. For example, concepts like heterogeneity, standardization, integration, and compatibility could be investigated to assess the factor of ‘interoperability’. As another example, legal aspects, political aspects, diffusion, ethical aspects, and policy of the organisation could be looked at for assessing the main or root factor, which is the ‘trust’ factor from ‘Organisation Dimension’. 

In term of compatibility and integration with other AAL models and frameworks, our AAL Adoption Diamond Framework is compatible and can be integrated with other relevant models and frameworks for a thorough assessment and investigation of one or more factors. For an example, TAM could be used with our framework to investigate the factor ‘user acceptance’ more profoundly. Method-wise, combination of evaluative approaches would ensure a mix of approaches that would be more comprehensive fitting the needs of a specific research or implementation project. A combination of frameworks and evaluation tools seem to be the go-to approach in the fast-paced field of AAL technology. Choukou et al. (2021) [66] have recently reviewed the assessment tools and approaches used to evaluate the acceptance of AAL technology and highlighted the need to combine evaluative approaches and subsequent tools to cover all the acceptability attributes of a given project, depending on its progress stage, in conformity with the most recent recommendations by ISO (2018) [67]. Therefore, the authors of this paper recommend using the Diamond Framework as a standalone model or in conjunction with their framework, and they advise users to base their decisions on their theoretical and pragmatic needs, as well as to use evaluation/analysis tools in a flexible manner that meets the needs of the attributes they seek.

### 4.4. Study Limitations

The thematic analysis performed in this study mainly focused on the extracted data related to the factors contributing to AAL technologies adoption. The limited data extracted from the articles were not sufficient to know how these factors were defined in some studies. Also, due to the heterogeneity in the included studies’ design and AAL technology types, it was not appropriate to compare the factors’ definitions. For this reason, we conducted an additional search in the grey literature and provided more details when required. While the Diamond Framework fills in a conceptual gap in AAL technology, we encourage further investigation and literature updates to reach standardization and increase the use of the Diamond Framework in empirical investigations.

## 5. Conclusions

AAL technologies have been poorly adopted and undervalued for delivering health care to the elderly. Our work led to developing the AAL Adoption Diamond Framework which is holistic and novel as it identifies four key dimensions: Human, Technology, Business, and Organisation, as well as nineteen factors. This framework could support stakeholders in recognizing the key factors contributing to AAL adoption. This comprehension is essential for informing the design of AAL technologies and hence improving the adoption and implementation of such technologies by elder people. In the future, the researchers aim to empirically test the framework and its capability for evaluating AAL technology adoption by different stakeholders (e.g., caregivers, health care providers, and decision-makers) in a Saudi healthcare context which also serves as a further validation and verification of these factors and their descriptions.

## Figures and Tables

**Table 1:** The inclusion and exclusion criteria.

Inclusion Criteria	Exclusion Criteria
It was categorized under those disciplines: Social Science, e-health and Health Informatics, Computer Science.	Articles which their focus was mainly on hardware architecture (engineering discipline) were excluded; however, no restrictions were made regarding the methodologies of studies (e.g., empirical quantitative or qualitative studies, and non-empirical studies such as systematic reviews were included).
It was published in the last ten years (2009 or later).	Articles published before 2009 were not included because AAL technologies are advancing rapidly, hence ensuring the inclusion of studies that reflect the recent advancement in the AAL domain.
Studies that targeted older adults (60 years and older as defined by the United Nations in 2013), and were written in English	Articles published in Arabic or other languages were not considered.

**Table 2:** The characteristics of the included studies.

Authors and Years	Types of Technologies				
	Ambient Assisted Living (AAL)	Smart Home (SH)	Assistive Robotics (AR)	Wearable and Mobile Devices (WMD)	e-T
Sun et al. (2009) [25]	✓				
Muñoz et al. (2011) [6]	✓				
Ding et al. (2011) [26]		✓			
Pogorelc et al. (2012) [27]	✓				
Wu et al. (2012) [28]			✓		
Grgurić, (2012) [29]	✓				
Chan et al. (2012) [30]				✓	
Paoli et al. (2012) [31]	✓				
Lê et al. (2012) [32]		✓			
Flandorfer, (2012) [33]			✓		
Balta-Ozkan et al. (2013) [9]		✓			
Portet et al. (2013) [34]		✓			
Berglin, (2013) [10]					✓
Ayala & Amor (2013) [7]	✓				
Khosla et al. (2013) [35]			✓		
Kim & Jeong (2013) [36]		✓			
Parker et al. (2013) [37]				✓	
Rashidi & Mihailidis (2013) [38]	✓				
Spitalewsky et al. (2013) [39]	✓				
Morris et al. (2013) [40]		✓			
AALIANCE2, (2014) [19]	✓				
Memon et al. (2014) [41]	✓				
Spasova & Iliev (2014) [42]	✓				
Wu et al. (2014) [43]			✓		
Hersh (2015) [44]			✓		
Jaschinski & Allouch (2015) [45]	✓				
Peruzzini & Germani (2015) [46]	✓				
Li et al. (2015) [47]	✓				
Dasios et al. (2015) [48]	✓				
Fletcher & Jensen (2015) [49]				✓	
Ni et al. (2015) [50]		✓			
Jacobsson et al. (2016) [51]		✓			
Al-Shaqi et al. (2016) [4]	✓				
Ariani et al. (2016) [5]			✓		
Wang et al. (2016) [52]				✓	
Wilson et al. (2017) [53]		✓			
Alsinglawi et al. (2017) [54]		✓			
Majumder et al. (2017) [55]		✓			
Halslwanter & Fitzpatrick (2017) [56]	✓				
Gonçalves et al.,(2018) [8]					✓
Do et al. (2018) [57]			✓		
Biermann et al. (2018) [58]	✓				
Pal et al. (2018) [59]		✓			
Carnemolla, (2018) [20]		✓			
Spann & Stewart (2018) [60]				✓	
Bozan & Berger (2019) [61]	✓				
Marikyan et al. (2019) [62]		✓			
Pal et al. (2019) [59]		✓			
El & Abtoy (2019) [63]	✓				
Grgurić et al. (2019) [64]	✓				
Wang et al. (2019) [65]	✓				

## Data Availability

Not applicable.

## References

[1] World Health Organisation Ageing and Health. https://www.who.int/news-room/fact-sheets/detail/ageing-and-health.

[2] Al-Aama T. (2011). Falls in the elderly: Spectrum and prevention. Can. Fam. Physician.

[3] Centers for Disease Control and Prevention Facts about Falls. https://www.cdc.gov/falls/facts.html#print.

[4] Al-Shaqi R., Mourshed M., Rezgui Y. (2016). Progress in ambient assisted systems for independent living by the elderly. Springerplus.

[5] Arni A., Vasvi K., Amir T.-K., Junhua L., Pradeep K.R. (2016). Challenges in Seniors Adopting Assistive Robots: A Systematic Review. Int. Technol. Manag. Rev..

[6] Muñoz A., Augusto J.C., Villa A., Blaya J.A.B. (2011). Design and evaluation of an ambient assisted living system based on an argumentative multi-agent system. Pers. Ubiquitous Comput..

[7] Ayala I., Amor M., Fuentes L. (2013). Self-configuring agents for ambient assisted living applications. Pers. Ubiquitous Comput..

[8] Gonçalves C., Ferreira da Silva A., Gomes J., Simoes R. (2018). Wearable E-Textile Technologies: A Review on Sensors, Actuators and Control Elements. Inventions.

[9] Balta-Ozkan N., Davidson R., Bicket M., Whitmarsh L. (2013). Social barriers to the adoption of smart homes. Energy Policy.

[10] Berglin L. (2013). Smart Textiles and Wearable Technology.

[11] Schomakers E.-M., Heek J.O.-v., Ziefle M. Playfully Assessing the Acceptance and Choice of Ambient Assisted Living Technologies by Older Adults. Proceedings of the ICT4AWE 2018: Information and Communication Technologies for Ageing Well and e-Health.

[12] Alsulami M.H., Atkins A.S., Campion R.J. Factors Influencing the Adoption of Ambient Assisted Living Technologies by Healthcare Providers in the Kingdom of Saudi Arabia. Staffordshire University. Proceedings of the AIT2S 2017: Advanced Information Technology, Services and Systems.

[13] Maranesi E., Amabili G., Cucchieri G., Bolognini S., Margaritini A., Bevilacqua R., Scataglini S., Imbesi S., Marques G. (2022). Understanding the Acceptance of IoT and Social Assistive Robotics for the Healthcare Sector: A Review of the Current User-Centred Applications for the Older Users. Internet of Things for Human-Centered Design: Application to Elderly Healthcare.

[14] Smit D., Eybers S. Towards a Socio-specific Artificial Intelligence Adoption Framework. Proceedings of the 43rd Conference of the South African Institute of Computer Scientists and Information Technologists.

[15] Davis F.D., Bagozzi R.P., Warshaw P.R. (1989). User Acceptance of Computer Technology: A Comparison of Two Theoretical Models. Manag. Sci..

[16] Taylor S., Todd P.A. (1995). Understanding Information Technology Usage: A Test of Competing Models. Inf. Syst. Res..

[17] Fishbein M., Ajzen I. (1977). Belief, Attitude, Intention, and Behavior: An Introduction to Theory and Research. Philos. Rhetor..

[18] Tornatzky L.G., Fleischer M., Chakrabarti A.K. (1990). The Processes of Technological Innovation, Issues in Organization and Management Series.

[19] AALIANCE2 Ambient Assisted Living Roadmap. http://www.aaliance2.eu/sites/default/files/AA2_WP2_D27_RM2_rev5.0.pdf.

[20] Carnemolla P. (2018). Ageing in place and the internet of things—How smart home technologies, the built environment and caregiving intersect. Vis. Eng..

[21] Tessarolo F., Petsani D., Conotter V., Nollo G., Conti G., Nikolaidou M., Onorati G., Bamidis P.D., Konstantinidis E.I. (2022). Developing ambient assisted living technologies exploiting potential of user-centred co-creation and agile methodology: The CAPTAIN project experience. J. Ambient. Intell. Humaniz. Comput..

[22] Liberati A., Altman D.G., Tetzlaff J., Mulrow C., Gøtzsche P.C., Ioannidis J.P., Clarke M., Devereaux P.J., Kleijnen J., Moher D. (2009). The PRISMA statement for reporting systematic reviews and meta-analyses of studies that evaluate health care interventions: Explanation and elaboration. PLoS Med..

[23] Page M.J., McKenzie J.E., Bossuyt P.M., Boutron I., Hoffmann T.C., Mulrow C.D., Shamseer L., Tetzlaff J.M., Akl E.A., Brennan S.E. (2021). The PRISMA 2020 statement: An updated guideline for reporting systematic reviews. Syst. Rev..

[24] Braun V., Clarke V. (2006). Using thematic analysis in psychology. Qual. Res. Psychol..

[25] Sun H., Florio V.D., Gui N., Blondia C. Promises and Challenges of Ambient Assisted Living Systems. Proceedings of the 2009 Sixth International Conference on Information Technology: New Generations.

[26] Ding D., Cooper R.A., Pasquina P.F., Fici-Pasquina L. (2011). Sensor technology for smart homes. Maturitas.

[27] Pogorelc B., Bosnić Z., Gams M. (2012). Automatic recognition of gait-related health problems in the elderly using machine learning. Multimed. Tools Appl..

[28] Wu Y.-H., Fassert C., Rigaud A.-S. (2012). Designing robots for the elderly: Appearance issue and beyond. Arch. Gerontol. Geriatr..

[29] Grgurić A. (2012). ICT Towards Elderly Independent Living.

[30] Chan M., Estève D., Fourniols J.-Y., Escriba C., Campo E. (2012). Smart wearable systems: Current status and future challenges. Artif. Intell. Med..

[31] Paoli R., Fernández-Luque F.J., Doménech G., Martínez F., Zapata J., Ruiz R. (2012). A system for ubiquitous fall monitoring at home via a wireless sensor network and a wearable mote. Expert Syst. Appl..

[32] Lê Q., Nguyen H.B., Barnett T. (2012). Smart Homes for Older People: Positive Aging in a Digital World. Future Internet.

[33] Flandorfer P. Drivers, Barriers and long-term Requirements of assistive Technologies supporting older Persons in living longer independently at Home: A systematic Review of European, US-American and Japanese Policy Papers and Assessment Studies. Proceedings of the European Population Conference.

[34] Portet F., Vacher M., Golanski C., Roux C., Meillon B. (2011). Design and evaluation of a smart home voice interface for the elderly: Acceptability and objection aspects. Pers. Ubiquitous Comput..

[35] Khosla R., Chu M.-T., Nguyen K. Affective Robot Enabled Capacity and Quality Improvement of Nursing Home Aged Care Services in Australia. Proceedings of the 2013 IEEE 37th Annual Computer Software and Applications Conference Workshops.

[36] Kim S.-C., Jeong Y.-S., Park S.-O. (2013). RFID-based indoor location tracking to ensure the safety of the elderly in smart home environments. Pers. Ubiquitous Comput..

[37] Parker S.J., Jessel S., Richardson J.E., Reid M.C. (2013). Older adults are mobile too!Identifying the barriers and facilitators to older adults’ use of mHealth for pain management. BMC Geriatr..

[38] Rashidi P., Mihailidis A. (2013). A survey on ambient-assisted living tools for older adults. IEEE J. Biomed. Health Inform..

[39] Spitalewsky K., Rochon J., Ganzinger M., Knaup P. (2013). Potential and requirements of IT for ambient assisted living technologies. Results of a Delphi study. Methods Inf. Med..

[40] Morris M.E., Adair B., Miller K.J., Ozanne E., Hansen R., Pearce A.J., Santamaria N., Viegas L., Long M., Said C.M. (2013). Smart-Home Technologies to Assist Older People to Live Well at Home. J. Aging Sci..

[41] Memon M., Wagner S.R., Pedersen C.F., Beevi F.H., Hansen F.O. (2014). Ambient assisted living healthcare frameworks, platforms, standards, and quality attributes. Sensors.

[42] Spasova V., Iliev I. (2014). A survey on automatic fall detection in the context of ambient assisted living systems. Int. J. Adv. Comput. Res..

[43] Wu Y.H., Wrobel J., Cornuet M., Kerhervé H., Damnée S., Rigaud A.S. (2014). Acceptance of an assistive robot in older adults: A mixed-method study of human-robot interaction over a 1-month period in the Living Lab setting. Clin. Interv. Aging.

[44] Hersh M. (2015). Overcoming Barriers and Increasing Independence—Service Robots for Elderly and Disabled People. Int. J. Adv. Robot. Syst..

[45] Jaschinski C., Allouch S.B. (2015). An extended view on benefits and barriers of ambient assisted living solutions. Adv. Life Sci..

[46] Peruzzini M., Germani M. (2016). Design of a service-oriented architecture for Ambient-assisted Living. Int. J. Agil. Syst. Manag..

[47] Li R., Lu B., McDonald-Maier K.D. (2015). Cognitive assisted living ambient system: A survey. Digit. Commun. Netw..

[48] Dasios A., Gavalas D., Pantziou G., Konstantopoulos C. (2015). Hands-On Experiences in Deploying Cost-Effective Ambient-Assisted Living Systems. Sensors.

[49] Fletcher J., Jensen R. (2015). Overcoming Barriers to Mobile Health Technology Use in the Aging Population: OJNI. J. Nurs. Inform..

[50] Ni Q., García Hernando A.B., De la Cruz I.P. (2015). The Elderly’s Independent Living in Smart Homes: A Characterization of Activities and Sensing Infrastructure Survey to Facilitate Services Development. Sensors.

[51] Jacobsson A., Boldt M., Carlsson B. (2016). A risk analysis of a smart home automation system. Future Gener. Comput. Syst..

[52] Wang J., Carroll D., Peck M., Myneni S., Gong Y. (2016). Mobile and Wearable Technology Needs for Aging in Place: Perspectives from Older Adults and Their Caregivers and Providers. Study Health Technol. Inform..

[53] Wilson C., Hargreaves T., Hauxwell-Baldwin R. (2017). Benefits and risks of smart home technologies. Energy Policy.

[54] Alsinglawi B., Nguyen Q.V., Gunawardana U., Maeder A., Simoff S. (2017). RFID Systems in Healthcare Settings and Activity of Daily Living in Smart Homes: A Review. E-Health Telecommun. Syst. Netw..

[55] Majumder S., Aghayi E., Noferesti M., Memarzadeh-Tehran H., Mondal T., Pang Z., Deen M.J. (2017). Smart Homes for Elderly Healthcare-Recent Advances and Research Challenges. Sensors.

[56] Hallewell Haslwanter J., Fitzpatrick G. (2017). The Development of Assistive Systems to Support Older People: Issues that Affect Success in Practice. Technologies.

[57] Do H.M., Pham M., Sheng W., Yang D., Liu M. (2018). RiSH: A robot-integrated smart home for elderly care. Robot. Auton. Syst..

[58] Biermann H., Offermann-van Heek J., Himmel S., Ziefle M. (2018). Ambient Assisted Living as Support for Aging in Place: Quantitative Users’ Acceptance Study on Ultrasonic Whistles. JMIR Aging.

[59] Pal D., Funilkul S., Vanijja V., Papasratorn B. (2018). Analyzing the Elderly Users’ Adoption of Smart-Home Services. IEEE Access.

[60] Spann A., Stewart E. (2018). Barriers and facilitators of older people’s mHealth usage: A qualitative review of older people’s views. Hum. Technol..

[61] Bozan K., Berger A. Revisiting the technology challenges and proposing enhancements in ambient assisted living for the elderly. Proceedings of the Annual Hawaii International Conference on System Sciences IEEE Computer Society.

[62] Marikyan D., Papagiannidis S., Alamanos E. (2019). A systematic review of the smart home literature: A user perspective. Technol. Forecast. Soc. Chang..

[63] El Murabet A., Abtoy A. (2019). Understanding the ambient Assisted Living systems: Concepts, architectural trends and challenges. Int. J. Open Inf. Technol..

[64] Grgurić A., Mošmondor M., Huljenić D. (2019). The SmartHabits: An Intelligent Privacy-Aware Home Care Assistance System. Sensors.

[65] Wang S., Bolling K., Mao W., Reichstadt J., Jeste D., Kim H.C., Nebeker C. (2019). Technology to Support Aging in Place: Older Adults’ Perspectives. Healthcare.

[66] Choukou M.A., Shortly T., Leclerc N., Freier D., Lessard G., Demers L., Auger C. (2021). Evaluating the acceptance of ambient assisted living technology (AALT) in rehabilitation: A scoping review. Int. J. Med. Inform..

[67] ISO (2018). Ergonomics of Human-System Interaction—Part 11: Usability: Definitions and Concepts.

